# Malaria Vectors in Ecologically Heterogeneous Localities of the Colombian Pacific Region

**DOI:** 10.1371/journal.pone.0103769

**Published:** 2014-08-04

**Authors:** Nelson Naranjo-Díaz, Mariano Altamiranda, Shirley Luckhart, Jan E. Conn, Margarita M. Correa

**Affiliations:** 1 Grupo de Microbiología Molecular, Escuela de Microbiología, Universidad de Antioquia UdeA, Medellín, Colombia; 2 Department of Medical Microbiology and Immunology, University of California Davis, Davis, California, United States of America; 3 Griffin Laboratory, Wadsworth Center, New York State Department of Health, Singerlands, New York, United States of America; Department of Biomedical Sciences, School of Public Health, State University of New York, Albany, New York, United States of America; Centro de Pesquisas René Rachou, Brazil

## Abstract

The Colombian Pacific region is second nationally in number of malaria cases reported. This zone presents great ecological heterogeneity and *Anopheles* species diversity. However, little is known about the current spatial and temporal distribution of vector species. This study, conducted in three ecologically different localities of the Pacific region, aimed to evaluate the composition and distribution of *Anopheles* species and characterize transmission intensity. A total of 4,016 *Anopheles* mosquitoes were collected representing seven species. The composition and dominant species differed in each locality. Three species were infected with malaria parasites: *Anopheles darlingi* and *An. calderoni* were infected with *Plasmodium falciparum* and *An. nuneztovari* with *Plasmodium vivax* VK210 and VK247. Annual EIRs varied from 3.5–7.2 infective bites per year. These results confirm the importance of the primary vector *An. nuneztovari* in areas disturbed by human interventions, of *An. darlingi* in deforested margins of humid tropical rainforest and *An. albimanus* and the suspected vector *An. calderoni* in areas impacted by urbanization and large-scale palm oil agriculture close to the coast. This constitutes the first report in the Colombia Pacific region of naturally infected *An. darlingi*, and in Colombia of naturally infected *An. calderoni.* Further studies should evaluate the epidemiological importance of *An. calderoni* in the Pacific region.

## Introduction

The Colombian Pacific (PAC) region is second in the number of national malaria cases reported [1], registering an average of 30% of the total cases [2]–[4] with the majority of cases due to infection with *Plasmodium falciparum* Welch (>60%) [1]. PAC is located in the biogeographic Chocó zone [5] and presents diverse ecological and climate conditions with five natural subregions [6] that are predicted to affect *Anopheles* vector species distribution and behavior [7].

Three species considered the main Colombian malaria vectors are present in the PAC region [7]–[9], with reports of natural *Plasmodium* infection for *Anopheles* (*Nyssorhynchus*) *nuneztovari* Gabaldon [10] and *Anopheles* (*Nys*.) *albimanus* Wiedemann [8]. Although *Anopheles* (*Nys*.) *darlingi* is considered the main vector in the Chocó rainforest [11], [12], there have been no published records of naturally infected *An. darlingi* in this area. *Anopheles (Kerteszia) neivai* Howard, Dyar & Knab, a species closely associated with forest bromeliads and mangroves [13], is a locally important vector in PAC and has been reported infected with *P. falciparum* [13] and *P. vivax* [8], [14]. Existing records also suggest an epidemiological association of *Anopheles* (*Anopheles*) *punctimacula* Dyar & Knab with malaria outbreaks in this area [15], [16]; this species was detected with salivary gland sporozoites of *P. vivax* in NW Colombia [17]. Various members of the Arribalzagia Series such as *An. punctimacula*, *Anopheles malefactor* Dyar & Knab, *Anopheles calderoni* Wilkerson and *Anopheles guarao* Anduze & Capdevielle, are characterized by a high degree of isomorphism that complicates their differentiation and accurate identification by morphological characters [18]–[20]. For example, in mosquito studies in PAC, *An. punctimacula* was the species recorded; however, *An. calderoni* was recently reported in various PAC localities, as supported by adult and larval diagnostic characters and mosquito barcoding [21]. In Piura Department in neighboring Peru, *An. calderoni* was infected with *P. vivax* and considered a locally important vector [22], [23]. Collectively, these observations suggest the need for further evaluation of the identity of these species and their involvement in malaria transmission.

Most vector biology studies in PAC have focused on distributions and parasite infection of the *Anopheles* species present, but little is known about variation in vector densities and transmission intensities at spatial and temporal scales. Therefore, a longitudinal survey was conducted from April 2009 to June 2010 in three ecologically diverse localities of the epidemiologically important PAC region to evaluate species composition and distributions at spatial and temporal levels and to determine important entomological parameters.

## Materials and Methods

### Study sites

**Figure 1:**
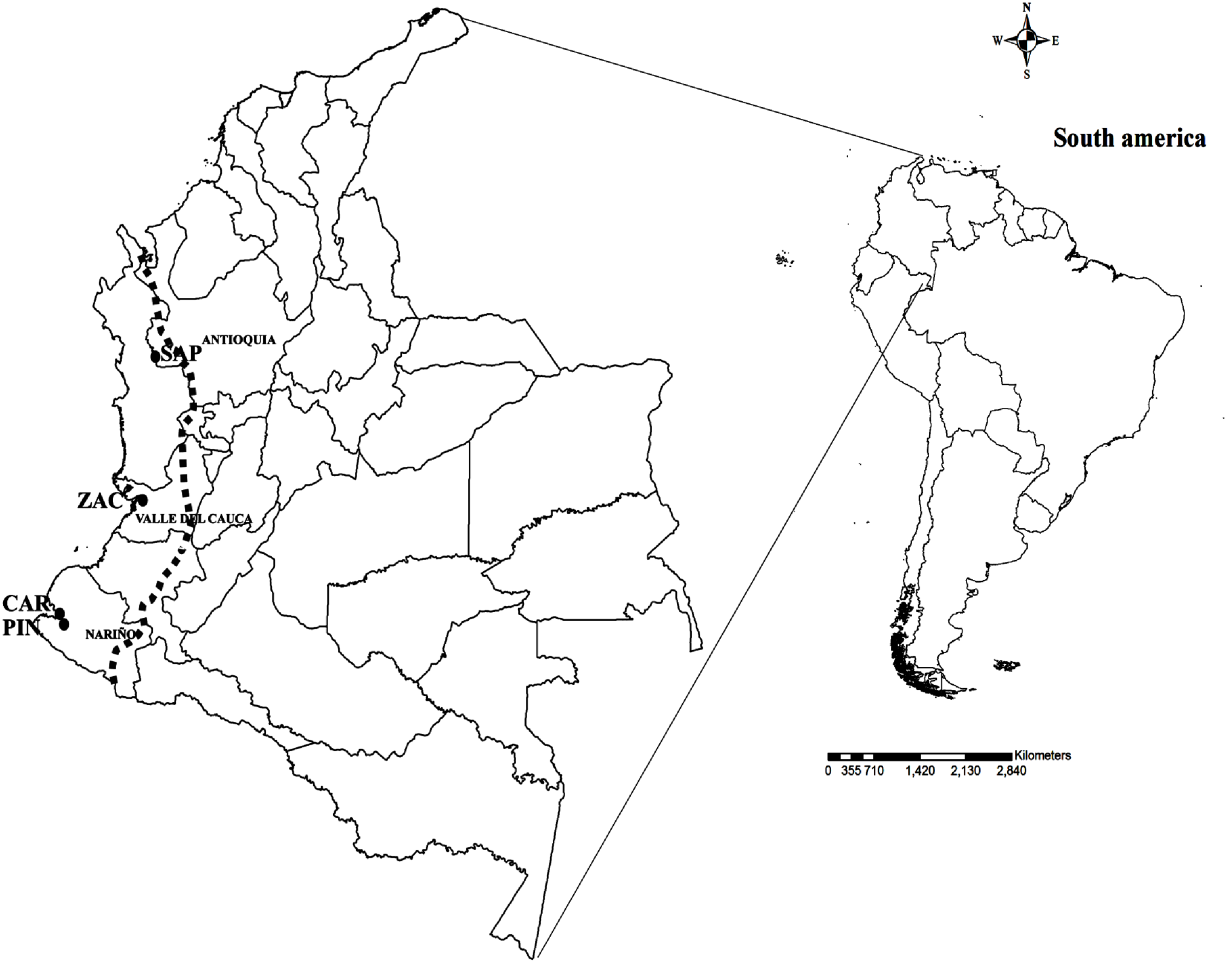
Mosquito collection sites in PAC region. San Antonio de Padua (SAP) in the municipality of Vigía del Fuerte, Antioquia Department; Zacarias (ZAC) in Buenaventura, Valle del Cauca Department and Carbonera-Pindales (CAR-PIN) in San Andres de Tumaco, Nariño Department. Spotted line defines Pacific Region boundaries.

**Figure 2:**
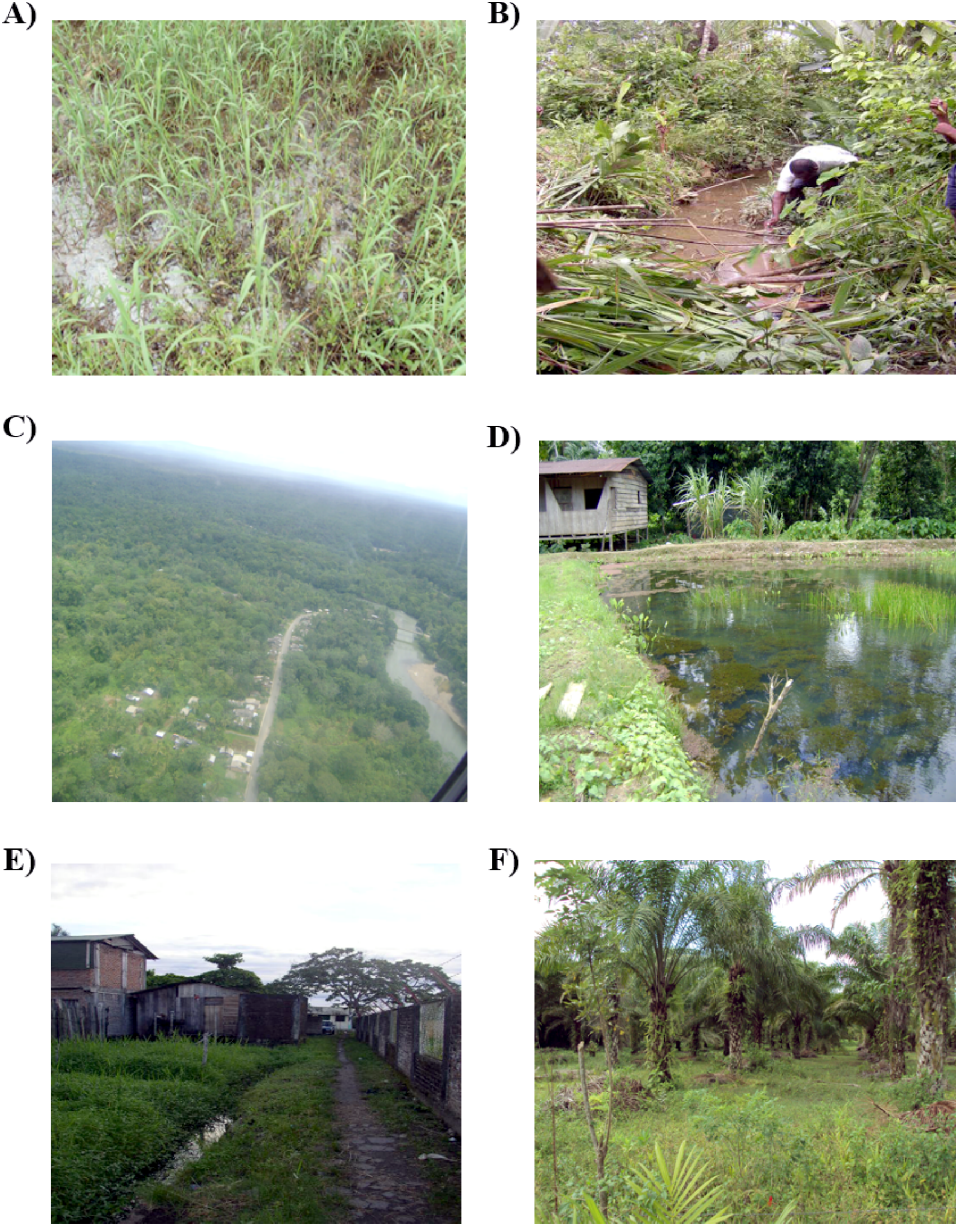
Characteristic of sampling sites. San Antionio de Padua locality (SAP): Larval habitats A) originated from rice cultivation. B) pond in the forest border. Zacarias (ZAC): C) ZAC settlement and nearby river used for material extraction. D) Larval habitat - fishpond. E) Carbonera (CAR): Larval habitat - drainage channel. F) Pindales (PIN): oil-palm agriculture.

Adult *Anopheles* were collected from three ecologically diverse malaria endemic localities of the PAC region (Table 1, Figure 1); they were San Antonio de Padua (SAP), Zacarias (ZAC), and Carbonera and Pindales (CAR-PIN). SAP is in the PAC natural subregion Alluvial Valleys of the Atrato and San Juan rivers which is characterized by tropical forest [6]. The main economic activities in SAP are fishing, small-scale agriculture, especially rice and logging that is displacing the forest borders (Figure 2A–B). ZAC is in the PAC Coastal Plains subregion characterized by flooded plains, lakes and swamps [6] and presents one of the highest levels of precipitation in the world (>5,000 mm annually), with rainfalls occurring most of the year [6]. In ZAC, deforestation for human settlements, river extraction of building materials and pisciculture are the most important activities (Figures 2C–D). CAR and PIN are separated by 9 km, and, like ZAC, are located in the Coastal Plains subregion. Both localities are heavily impacted by anthropic changes. CAR is located approximately 3 km from the coast and is a periurban neighborhood of the municipality of San Andres de Tumaco, where urbanization has replaced wetlands and mangroves (Figure 2E). The first collection for these localities was performed in CAR. However, because of problems of public order, the following three collections were completed in PIN, located approximately 11 km from the coast and characterized by large-scale palm oil agriculture (Figure 2F).

**Table 1:** Data on abundance, HBR, IR and EIR for the *Anopheles* species collected.

Locality/	Year	Species	*n* (%)	HBR	IR %	Annual EIR
Abbreviation/	Collection month				(CI)	
Municipality/	(Number of days)					
Department						
**San Antonio de Padua**	2009	*An. darlingi*	1,079 (99.8)	89.9		
**(SAP)**	April (4)	*An. nuneztovari*	2 (0.2)	0.2		
Vigía del Fuerte						
Antioquia	2009	*An. darlingi*	219 (99.1)	9		
06° 17′N 76°45′W	November (6)	*An. punctimacula*	2 (0.9)	0.08		
						
	2010	***An. darlingi***	336 (90.8)	13.8	0.036 *Pf* a	5.2
	**March (6)**	*An. nuneztovari*	32 (8.7)	1.3	(0.001–0.203)	
		*An. punctimacula*	2 (0.5)	0.08		
						
	2010	*An. darlingi*	1,104 (99.9)	46.1		
	June (5)	*An. costai/forattinii*	1 (0.1)	0.05		
**Zacarias**	2009	*An. nuneztovari*	100 (96.2)	4.6		
**(ZAC)**	April (5)	*An. neivai*	4 (3.8)	0.2		
Buenaventura						
Valle del Cauca	2009	*An. nuneztovari*	116 (98.3)	4.3		
03°49′N 76°59′W	August (6)	*An. neivai*	2 (1.7)	0.08		
						
	2009	*An. nuneztovari*	69 (93.2)	2.7		
	October (6)	*An. neivai*	5 (6.8)	0.2		
						
	2010	***An. nuneztovari***	220 (100)	8.6	0.203 *Pv* K210^a^	7.2
	**February (6)**				0.203 *Pv* K247^b^	
					(0.005–1.127)	
**Carbonera**	2009 †	*An. albimanus*	109 (97.3)	4.4		
01°46′N 78°47′W	June (5)	*An. calderoni*	3 (2.7)	0.1		
**Pindales**						
**(CAR-PIN)**	2009	*An. calderoni*	42 (93.3)	1.7		
San Andres de Tumaco	October (6)	*An. albimanus*	3 (6.7)	0.1		
Nariño						
01°37′N 78°44′W	2010	*An. calderoni*	90 (58.1)	3.5		
	January (6)	*An. albimanus*	65 (41.9)	2.6		
						
	2010	***An. calderoni***	400 (97.5)	14.7	0.194 *Pf* a	3.5
	**April (6)**	*An. albimanus*	10 (2.5)	0.4	(0.005–1.079)	

*n*: total number of *Anopheles* collected by period. %: abundance relative expressed in percentage. HBR: human biting rate for each species as mosquito bites/person/night for each date and site. IR: infection rate as percentage of infected specimens of the total collected,

a determined by a positive result on the first ELISA of mosquito pools and nested PCR of individual abdomens of positive pools,

b determined by the first and second positive pools of ELISA and nested PCR of individual abdomens of positive pools. CI: IR confidence interval. *Pv*: *Plasmodium vivax*, *Pf*: *Plasmodium falciparum*. EIR: entomological inoculation rate or number of potential infective mosquito bites per species per year. Boldfaced: collection period and name of the species with infected mosquitoes.

† First collection period conducted in Carbonera locality.

### Mosquito collection

Each locality was visited four times, on average once every three months, from April 2009 to June 2010. Mosquitoes were collected indoors and outdoors (within ∼10 m of the house) by human landing catch under a protocol and informed consent agreement approved by a University of Antioquia Institutional Review Board (Comité de Bioética, Sede de Investigación Universitaria, CBEIH-SIU, approval number 07-41-082). Collections were conducted by two people in each setting, for five days, from 18∶00 to 24∶00 h and one additional night from 18∶00 to 06∶00 h. Adult mosquitoes were identified using morphology-based keys [24]–[26]. Species presenting difficulties during morphological identification were confirmed by PCR-RFLP-ITS2 [27]–[29] and specimens *An. calderoni* by *COI* barcoding [30] using primers by Folmer et al. [31] and standardized conditions [32]. In addition, potential larval habitats within approximately 1 km of adult collection sites were inspected to identify those positive for immature stages and third and fourth instar larvae were identified. Mosquito vouchers were deposited in the collection of “Grupo de Microbiología Molecular, Universidad de Antioquia”, and *An. calderoni COI* sequences were deposited in GenBank (Accesion numbers KF698816–KF698832).

### Detection of natural *Plasmodium* infection

Natural infections with *P. falciparum* and *P. vivax* (VK210 and VK247) were determined by ELISA [8], [33], [34], using pools of heads and thoraces of up to five specimens of the same species. The ELISA was used as a screening test to evaluate a large number of mosquitoes. Positive pools in the ELISA were confirmed by a second ELISA. Nested genus specific PCR using DNA extracted from individual abdomens was performed to confirm ELISA results and to incriminate the individual specimen infected [35].

### Data analysis

Infection and entomological risk parameter analyses were described previously [36]. Briefly, the infection rate (IR) was calculated as the percentage of *Plasmodium* positive mosquitoes out of the total number of mosquitoes analyzed by species and locality. The human biting rate (HBR) was expressed as the average of female *Anopheles* bites per person per night. The annual EIR for each site corresponds to the number of infective bites that a person may receive in one year. Spearman’s correlation coefficient in the SPSS program, version 18 (SPSS Inc., Chicago, IL) was used to estimate the relationships between rainfall data of the previous month to a collection and mosquito abundance; the monthly rainfall records for each locality were obtained from the Instituto de Hidrología, Meteorología y Estudios Ambientales (IDEAM).

## Results

### Mosquito abundance

**Figure 3:**
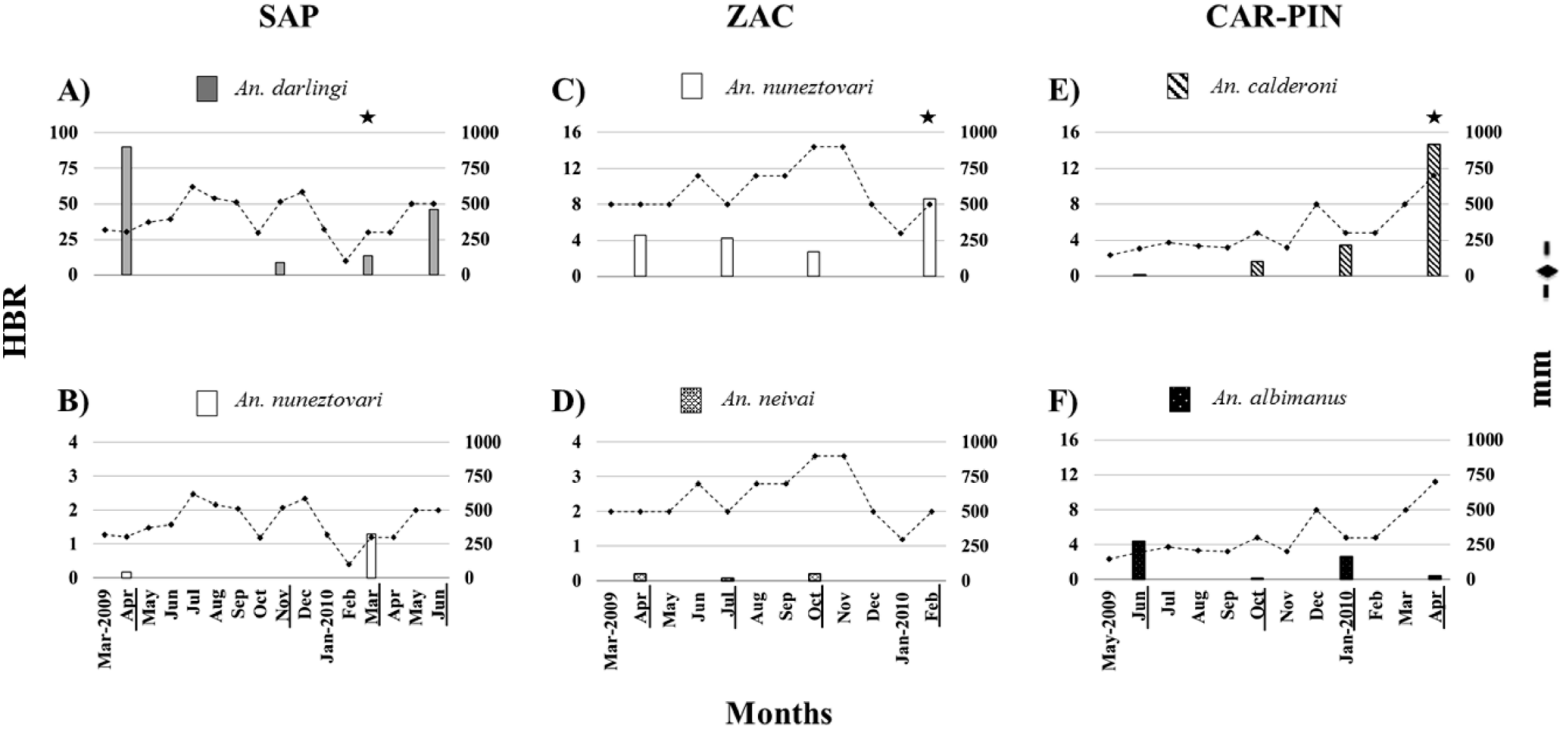
Relative abundance of the predominant species in relation to rainfall. Localities: San Antonio de Padua (SAP), Zacarias (ZAC), Carbonera-Pindales (CAR-PIN). Left axis: human biting rate (HBR) or number of mosquitoes/person/night, right axis: rainfall expressed in mm. The underlined months indicate collection periods and ★ periods when infected *Anopheles* were detected.

A total of 4,015 *Anopheles* mosquitoes corresponding to seven species were collected during 467 h of sampling (Table 1). There was not a significant (*p*>0.05) correlation between mosquito abundance and rainfall for any species. Species composition and dominance varied in each locality. In SAP, *An. darlingi* dominated (98.6%), and its highest abundance was observed during a period of moderate rainfall (Figure 3A). The main vector, *An. nuneztovari* was collected in low abundance in two samplings coinciding with the increase of rainfall in this locality (Figure 3B). *Anopheles punctimacula* was detected in low abundance in two consecutive collections (Table 1), with peak abundance at the beginning of the rainy season (Table 1, Figure 3B). *Anopheles costai/forattinii* was also collected by human biting but only in the last sampling period. *Anopheles*-positive larval habitats were rice irrigation channels and small ponds near forest borders, both sustained by water from the Atrato’s river (Figure 2A and 2B, respectively). The species detected in these larval habitats was *An. darlingi*. In one pond near the forest border, *An. malefactor*, a species not detected as an adult, co-occurred with *An. darlingi*.

In ZAC, *An. nuneztovari* was the dominant species (90%), detected in all collections; peak abundance was at the onset of a rainy period, when it was the only species collected (Table 1, Figure 3C). *Anopheles neivai* was only collected in this PAC locality, during the three initial samplings, always in low abundance. It was not detected in the fourth collection when there was a decrease in rainfall (average 900 to 300 mm) (Table 1, Figure 3D). The *Anopheles*-positive larval habitats in ZAC were fishponds (Figure 2D), swamps and temporal flooded ponds and the only larvae collected corresponded to *An. nuneztovari;* bromeliads were not sampled.

The dominant species in CAR was *An. albimanus* (97.3%), and in PIN, *An. calderoni* (87.21%). In the three collections in PIN, *An. calderoni* always outnumbered *An. albimanus* and its peak abundance corresponded to the highest rainy period in this locality (Table 1, Figure 3E). In the analyses of species distribution and peak abundance performed independently or together for specimens from CAR and PIN there were no significant differences, therefore these localities were presented as one (CAR-PIN). In CAR, *An. albimanus*-positive larval habitats were wetland drainage channels near houses (Figure 2E), and in PIN, irrigation channels of oil-palm agriculture (Figure 2F), and water wells where *An. albimanus* larvae were detected.

The total number of *Anopheles* and individual species collected did not show normal distributions (All: Kolmogorov-Smirnov Z = 4.3 *p*<0.001), except for *An. neivai* (Z = 1.96 *p*>0.05). In SAP, the number of *An. darlingi* ranged between 0 and 452 (Mean = 121.24, SD±121.26), *An. nuneztovari* between 0 and 23 (Mean = 1.62, SD±5.05), and *An. punctimacula* between 0 and 2 (Mean = 0.2, SD±0.51). In ZAC, *An. nuneztovari* ranged between 6 and 50 (Mean = 20.17, SD±11.28) and *An. neivai* between 0 and 3 (Mean = 0.5, SD±0.84). In CAR-PIN, *An. calderoni* ranged between 0 and 103 (Mean = 20.78, SD±28.67) and *An. albimanus* between 0 and 30 (Mean = 7.91, SD±9.97).

### Biting activity peak

**Figure 4:**
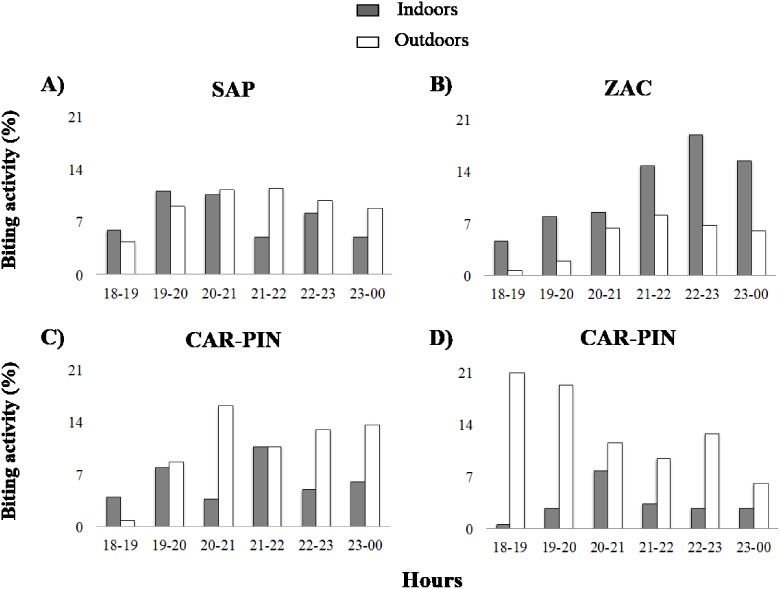
*Anopheles* biting activity. Biting activity is expressed as percentage of bites per human per hour. A. *An. darlingi* in San Antonio de Padua (SAP), B. *An. nuneztovari* in Zacarias (ZAC), C. *An. calderoni* in Carbonera-Pindales (CAR-PIN), D. *An. albimanus* in Carbonera-Pindales (CAR-PIN).

Mosquito biting activity, expressed as the mean proportion of mosquitoes collected per hour and per site, from 18∶00–24∶00 h, recorded for the most abundant species, varied by species (Figure 4). In SAP, *An. darlingi* showed a slight preference for biting outdoors, mostly after 20 h (61.3%) (Figure 4A), although no significant differences were detected (*t* = −1.61 *p*>0.05, *n* = 44) (not shown); its biting peak was between 19∶00–22∶00 h (Figure 3A). In ZAC, *An. nuneztovari* showed significant endophagic activity (*t* = 3.95 *p*<0.05, *n* = 23), with a main peak between 21∶00–23∶00 h (Figure 4B). In CAR-PIN, *An. calderoni* did not show a significant biting preference (*t* = −1.27 *p*>0.05, *n* = 23), the highest biting peak was between 20∶00–24∶00 h (Figure 4C). In contrast, *An. albimanus* showed a significant preference for biting outdoors (*t* = −2.4 *p*<0.05, *n* = 23), and was more active after sunset with its highest peak from 18∶00–21∶00 h (Fig. 4D).

### Human biting rate (HBR)

During the collection of April 2009 in SAP, *An. darlingi* exhibited the highest HBR among all species collected in any locality, 89.9 bites/person/night (b/p/n; Table 1). HBRs for *An. nuneztovari* were less than 10% of the highest HBR for *An. darlingi*; for example in ZAC this ranged between 2.7 and 8.6 b/p/n; in SAP, HBRs were between 0.2 and 1.3 b/p/n (Table 1). Particularly, *An. nuneztovari* was not detected in CAR-PIN where *An. calderoni* and *An. albimanus* were the only species collected in the four visits; furthermore, these species were exclusive to CAR-PIN. *Anopheles calderoni* exhibited its highest HBR in April 2010 (14.7 b/p/n), and *An. albimanus* in June 2009 collection (4.4 b/p/n) (Table 1). In general in PAC, species at low densities exhibited HBRs ≤0.2 b/p/n (Table 1).

### Infectivity and entomological inoculation rates

Four infected specimens were detected in the three PAC localities (Table 1). *Anopheles darlingi* in SAP and *An. calderoni* in CAR-PIN were infected with *P. falciparum* (IR = 0.036% and 0.194%, respectively). In ZAC, two *An. nuneztovari* were infected, one with *P. vivax* VK247 and the other with *P. vivax* VK210 (IR = 0.203% each). The annual EIR ranged from 3.5 infective bites per year (CAR-PIN) to 7.2 infective bites per year (ZAC), the highest recorded for this study (Table 1). Transmission intensity peaks or periods where *Anopheles* mosquitoes were detected infected with *Plasmodium* usually corresponded to low rainfall periods, except when *An. calderoni* was detected infected in PIN, during a rainy period (Table 1, Figure 3), that was also the period of its peak abundance.

## Discussion

*Anopheles* species dominance and abundance are influenced by environmental factors such as rainfall, land cover and topography [37]–[39]. In this longitudinal study in ecologically different localities of the malaria epidemiologically important PAC region, the influence of rainfall on *Anopheles* species abundance and diversity was analyzed and the main ecological characteristics of the localities described. In addition, the occurrence of the three main Colombia malaria vectors, *An. darlingi*, *An. nuneztovari* and *An. albimanus* was confirmed in PAC. However, the distribution and importance of these species as malaria vectors varied among localities.

For San Antonio de Padua locality, in a subregion characterized by tropical forest [6], *An. darlingi* was the most abundant and dominant species. In this locality *An. darlingi*-positive breeding sites were flooded, deforested forest margins and terrains used by the villagers for personal rice cultivation. Such ecological conditions and type of larval habitats favor the presence of this important vector [40]–[42]; i.e., in the Peruvian Amazon, *An. darlingi* adults dominate in deforested environments [43]. In SAP, the highest HBR for *An. darlingi* was during a moderate rainy period, but it is notable that this locality is characterized by persistent rain throughout the year. The HBR indicates that a person in SAP might receive 2,697 *An. darlingi* bites in a month, suggesting a higher likelihood of malaria transmission by this important vector. In a NW Colombian endemic area where malaria incidence is twice that of PAC, *An. darlingi* entomological parameters varied according to the locality and higher HBRs were observed at the beginning or end of rainy periods [36]. Data from this and other studies suggest that rainfall is a key factor affecting *An. darlingi* presence and dominance. For example, high HBRs have been correlated with low rainfall in Brazil [44], [45] and Venezuela [46], but also with rainy periods in the Amazonian forest of French Guiana [47] and Brazil [48]. These different responses to rainfall regimes combined with *An. darlingi*-positive larval habitats that had specific ecological conditions, suggest the importance of land cover [43], [49] and probable interaction effects among rainfall, ecological and land cover parameters on abundance and distribution of this main malaria vector in endemic regions of Colombia.

Annual EIR indicated that one person may receive one *An. darlingi* infective bite every 2.3 months. This EIR was higher than previously detected for this vector in the most important Colombian malaria endemic region, the Urabá Bajo Cauca and Alto Sinú (UCS) [36], indicating that *An. darlingi* is also important in malaria transmission in impacted habitats near rainforest. The infected *An. darlingi* specimen was detected when the rains were increasing after very low rainfall, in agreement with reports of high malaria transmission in Colombia, usually related to low or moderate rainfall periods [50]. In SAP, *An. darlingi* showed a biting peak between 19∶00–22∶00 h with a slight tendency for biting outdoors after 20 h. This behavior would be expected to increase infection risk since residents are active outdoors after work (N. Naranjo, personal communication), increasing human-vector contact [46]–[48].

In contrast, in SAP, the main Colombian vector *An. nuneztovari* was detected in low abundance, with HBRs ≤1. The highest density for this species was observed at the beginning of a rainy period when larval habitats are more stable compared to during heavy rainfall [51]. In Colombia, *An. nuneztovari* is characterized as being more tolerant of human impacted zones than *An. darlingi*, and it was previously detected in higher abundances in localities of the epidemiologically important UCS, NW Colombia [36], [52]. Also in SAP, *An. punctimacula*, a species of local importance, was present when the rain began to increase and showed even lower HBR values than *An. nuneztovari*. The presence of *An. punctimacula* during periods of low rains was also observed in NW Colombian localities [36], and its low abundance may be related to the collection methodology used in relation to its reported zoophilic tendency [53], [54].

In ZAC, in the Coastal Plains subregion of central PAC, *An. nuneztovari* and *An. neivai* were the only anthropophilic species detected. This locality is characterized by flooded plains and strongly influenced by deforestation and pisciculture. These human activities provide optimal larval habitats for *An. nuneztovari* [48], the dominant species during the entire sampling in ZAC. However, *An. nuneztovari* abundances were lower during the most intense rainfall period, coinciding with a decrease of *Anopheles*-positive larval habitats. Rainfall in this region is very high, with 6,980 mm annual average [6], which may cause flooding of the breeding sites affecting this species’ abundance. HBRs for *An. nuneztovari* ranged from 2.7 to 8.6 bites per person per night. Even lower HBRs have been previously reported for this species in Buenaventura municipality in PAC [55] and in other malaria endemic Colombian regions [36], [52], [56]; regardless of these observations, *An. nuneztovari* has previously been incriminated as a vector [36], [52].

In this study, the highest EIR was registered in ZAC for *An. nuneztovari* (7.5 infective bites per year), indicating that a person may receive one infective bite every one and a half months. This information, the fact that the two infected *An. nuneztovari* were detected in the period of its maximum abundance, together with the significant preference for biting indoors, suggest an increase in malaria risk for humans when they are resting at home (21∶00 to 24∶00 h). This information is of importance to reduce exposure and risk, especially inside the house, for example by the use of insecticide-treated mosquito nets, one of the main tools for vector control [57]. Various characteristics in ZAC favor the presence of *An. neivai*, a local vector in PAC [13], [58]. The presence of this species has been correlated to high humidity, lowland tropical forest and a variety of epiphyte plants that serve as its breeding sites [13]. However, in ZAC *An. neivai* was detected in low abundance (HBRs <1). It is possible that in-progress deforestation in this locality is reducing or eliminating preferred breeding sites of *An. neivai*. The low HBRs detected for *An. neivai* are similar to those previously reported in PAC for disturbed areas, compared with sylvatic conditions [7].

In CAR-PIN, localities impacted by anthropic changes, *An. albimanus* and *An. calderoni* were the only anthropophilic species detected. In particular, the dominant species in CAR was *An. albimanus* whereas in PIN it was *An. calderoni*. These differences may be related to their preferred larval habitats [7], [59], [60]. In CAR, mangroves and wetland drainage channels provide appropriate breeding sites for *An. albimanus*. The highest HBR for *An. albimanus* in CAR may be influenced by the proximity of this locality to the coast. Previous reports also show HBRs for *An. albimanus* increasing with proximity to the coast because of availability of its preferred larval habitats [7], [55]. Variation in *An. albimanus* abundance was not related to rainfall; however, HBRs decreased with increasing rainfall, and probably floods affected the stability of larval habitats. In PIN, small irrigation channels of oil-palm agriculture and wells favored the dominance of *An. calderoni*, previously associated with extensive palm agriculture near the coast [20], [61]. The highest HBR for *An. calderoni* was observed during the period of heaviest rainfall. Comparable HBRs have been reported for *An. calderoni* in other PAC localities, also during rainfall periods [62]. In PIN, high human-vector contact occurs from 18∶00 to 24∶00 h, with *An. albimanus* more active around sunset followed by *An. calderoni* from 21∶00 h. This specific information should help direct local vector control measures. In PIN, the annual EIR indicated that a person might receive one *An. calderoni* infective bite every three months. This species was recently confirmed in Buga, a PAC municipality, where it was initially identified as *An. punctimacula* [21], a species of local importance in some PAC localities [58]. However, *An. calderoni* has not yet been incriminated as a vector in Colombia, although it is of some importance in Peru [22], [23]. In this study, *An. calderoni* was detected infected with *P. falciparum* by the first ELISA and nested PCR but could not be confirmed by the second ELISA [63]. Given the difficulties still encountered in the tests to determine parasite infected mosquitoes, this result is reported here according to recommendation of defining a positive infected mosquito with the result of at least two positive tests [63]. Nevertheless, the status of *An. calderoni* as a local vector in PAC should be further evaluated. Even though *An. albimanus* is considered the main malaria vector in coastal PAC [8], [9], [59], it was not detected infected by *Plasmodium* spp. The predominant exophilic behavior of these two anthropophilic species suggest that application of residual insecticides on the outside walls of houses may be appropriate vector control.

## Conclusions

The ecological heterogeneity of the PAC localities was reflected in the variation of *Anopheles* species dominating in each one. Of concern is the fact that these species are among the main Colombian malaria vectors. Their importance in malaria transmission in different human impacted settings was confirmed. Infected *An. darlingi* was detected in deforested margins of humid tropical forest and *An. nuneztovari* in an area affected by various human interventions. The main PAC vector *An. albimanus* and the suspected vector *An. calderoni* were in areas impacted by urbanization and large-scale palm oil agriculture. Knowledge of their contribution to local malaria transmission and their spatial/temporal distributions helps to understand the dynamics of malaria transmission in this region and will provide the basis for the design and evaluation of more focused control measures.
